# Calibration and Deployment of a Fiber-Optic Sensing System for Monitoring Debris Flows

**DOI:** 10.3390/s120505835

**Published:** 2012-05-07

**Authors:** Ching-Jer Huang, Chung-Ray Chu, Tsung-Mo Tien, Hsiao-Yuen Yin, Ping-Sen Chen

**Affiliations:** 1 Department of Hydraulic and Ocean Engineering, National Cheng Kung University, No.1 University Road, Tainan, 70101, Taiwan; E-Mails: n8896106@mail.ncku.edu.tw (C.-R.C.); tmtien@gmail.com (T.-M.T.); 2 Monitoring and Management Division Soil and Water Conservation Bureau, Council of Agriculture, Nantou, 54044, Taiwan; E-Mails: sammya@mail.swcb.gov.tw (H.-Y.Y.); a670341@swcb.gov.tw (P.-S.C.)

**Keywords:** fiber-optic sensing system, FBG accelerometer, debris flow, ground vibration

## Abstract

This work presents a novel fiber-optic sensing system, capable of monitoring debris flows or other natural hazards that produce ground vibrations. The proposed sensing system comprises a demodulator (BraggSCOPE, FS5500), which includes a broadband light source and a data logger, a four-port coupler and four Fiber Bragg Grating (FBG) accelerometers. Based on field tests, the performance of the proposed fiber-optic sensing system is compared with that of a conventional sensing system that includes a geophone or a microphone. Following confirmation of the reliability of the proposed sensing system, the fiber-optic sensing systems are deployed along the Ai-Yu-Zi and Chu-Shui Creeks in Nautou County of central Taiwan for monitoring debris flows. Sensitivity test of the deployed fiber-optic sensing system along the creek banks is also performed. Analysis results of the seismic data recorded by the systems reveal in detail the frequency characteristics of the artificially generated ground vibrations. Results of this study demonstrate that the proposed fiber-optic sensing system is highly promising for use in monitoring natural disasters that generate ground vibrations.

## 1. Introduction

Debris flow is a rapid, gravity-induced flow of mixture of rocks, mud, and water [1,2]. Debris flows have the following characteristics: the front resembles a bore and the largest rocks accumulate there; the flow following the forefront appears as a mudflow with a slowly decreasing discharge; and the flow is accompanied by loud noise and ground vibration [3]. Monitoring ground vibrations, also referred to as seismic signals, is accepted as a reliable means of detecting such natural hazards [4,5].

Seismic signals caused by various natural hazards, e.g., earthquakes, landslides, debris flows, rock falls, snow avalanches, and pyroclastic flows are characterized by their frequency ranges and amplitudes. Suriñach *et al.* [6] investigated the seismic data produced by various earthquakes (*i.e.*, local, regional, and teleseism), a landslide, and artificially triggered snow avalanches. According to their results, frequency ranges of seismic signals produced by a local earthquake (52.8 km), regional earthquake (228.2 km) and teleseism (6,931.6 km) are 1–50 Hz, 1–12 Hz, and 0.1–1 Hz, respectively. While analyzing the seismic data excited by several pyroclastic flows occurring at the Unzen volcano in Kyushu, Japan, Uhira and Yamasata [7] found that the seismic waves contain low frequency components (0.5–10 Hz). Huang *et al.* [4] described the detection of debris flows at Ai-Yu-Zi Creek, (Nantou, Taiwan) using geophones (GS-20DX) to monitor ground vibrations produced by these debris flows. Their results indicate that when the main front was closest to the sensor, the frequency spectrum covered a wide range, from 10 to 250 Hz. The above studies demonstrate that selecting sensors to detect different natural hazards requires careful attention to the operating frequency range of sensors.

Many investigators have studied ground vibrations produced by debris flows [4,8–19] by using various types of sensors, including seismometers, geophones, microphones, and accelerometers. Of these sensors, geophones are most widely installed in systems for monitoring debris flows. However, ground tremors generated by debris flows are markedly smaller than those caused by earthquakes, and also have a higher range of frequencies. Attenuation of a seismic wave depends on its frequency. A high frequency is associated with a high spatial decay rate [20]. Therefore, debris flow tremors can only be detected over a relatively short distance. Although installing sensors close to the origins of debris flows can overcome this difficulty, the necessary use of long cables causes strong signal attenuation and transmission uncertainty in mountainous regions. While attempting to resolve the difficulty of long cable, recent works have developed wireless sensor networks (WSN) to monitor debris flows [21] and landslides [22].

Fiber-optic sensors have been developed recently for detecting various physical signals, including strain, temperature, and acceleration [23]. Owing to their light weight, immunity to electromagnetic interference, high sensitivity and extremely low optical loss, fiber-optic sensors are promising for use in monitoring debris flows or similar natural disasters, which usually occur in mountainous regions far from available electricity sources. Fiber-optic sensors use fibers to transmit light signals. Light waves generated by a light source are guided by an optical fiber to the measurement area. Differences in the physical variables, including strain, acceleration and temperature, can affect the light characteristics. Hence, changes in physical variables can be determined by demodulating the variation in light characteristics. Despite their use for monitoring in various fields, fiber-optic sensors have seldom been applied to monitor debris flows or other related natural disasters. Pei *et al.* [24] developed two fiber-optic sensing systems for monitoring landslides and debris flows. Their systems monitored debris flows by installing two steel pipes on the trench bank and then fixing a metal net crossing the section of the trench. FBG strainometers were glued at the bottom of the pipes to detect the pipe deformation induced by debris flows.

In this work, commercial fiber optical products, such as a FBG (Fiber Bragg Grating) accelerometer, coupler, and demodulator, are combined to set up a fiber-optic sensing system for monitoring ground vibrations generated by debris flows. Based on field tests, the performance of the proposed fiber-optic sensing system is compared with those of conventional systems using geophones or microphones. Following confirmation of the feasibility of the proposed system, fiber-optic sensing systems are deployed along Ai-Yu-Zi Creek and Chu-Shui Creek in Nautou County (Central Taiwan) for monitoring debris flows.

Following a brief review of the principles of the FBG accelerometer and the multiplexing method, this work presents a novel fiber-optic sensing system for monitoring debris flows. Next, based on field tests, the SNR values of the proposed system are compared with those of monitoring systems using a geophone or a microphone. Finally, the sensitivity of the proposed fiber-optic sensing system along creek banks is examined via further field tests.

## 2. Fiber-Optic Sensing Techniques and Multiplexing Method

**Figure 1.**
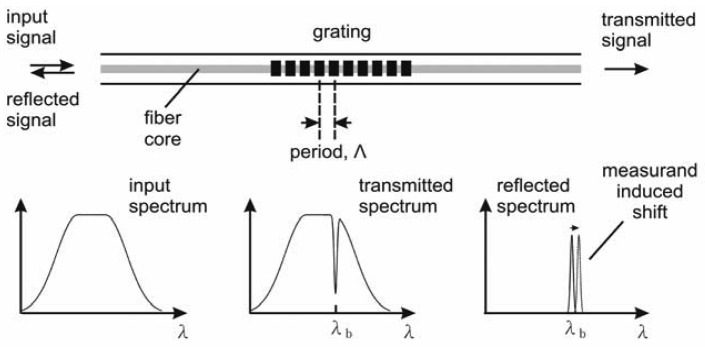
Mechanism of a Bragg grating-based sensing system [27].

An FBG sensor utilizes the variation of the periodic refraction index in a fiber section to reflect the light of a unique wavelength from a broadband light source [25]. The period of the grating structure is called the grating pitch, Λ (Figure 1). When broadband light is incident on the grating, a narrowband component, or a spectral slice, is reflected back at the Bragg resonance wavelength [26]. This wavelength is determined by:

(1) $$\lambda_{B}=2n\Lambda$$

where *n* is the effective index of the core. The Bragg resonance wavelength is shifted in response to an applied mechanical field as the effective index and the grating period vary with the strain and temperature. The shift in Bragg wavelength caused by changes in strain and temperature is given by Kersey *et al.* [27]:

(2) $$\frac{\Delta \lambda_{B}}{\lambda_{B}}=\left\{1-(\frac{n^{2}}{2})[P_{12}-\nu (P_{11}+P_{12})]\right\}\varepsilon +[\alpha +\frac{\left(dn/dT\right)}{n}]\Delta T$$

where *ε* is the applied strain; Δ*T* is the temperature change; *P*_ij_ are Pockel's (piezo) coefficients of the stress-optic tensor; *ν* is Poisson's ratio, and *α* is the coefficient of thermal expansion of the fiber material.

A debris flow normally lasts from several seconds to a few minutes. During this short period, the temperature varies only slightly and can thus be ignored. Therefore, during monitoring of debris flows when using the FBG sensor, the thermal term can be neglected and Equation (2) simplified to:

(3) $$\frac{\Delta \lambda_{B}}{\lambda_{B}}=P_{e}\varepsilon$$

where *P_e_* denotes the elasto-optic coefficient, which is a constant calculated by the strain term of Equation (2)

**Figure 2.**
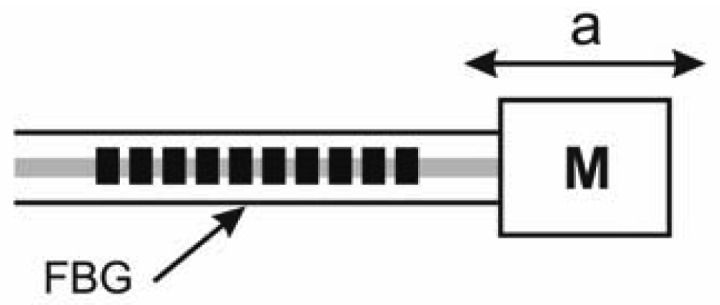
Diagram of a simple FBG accelerometer.

Numerous methods have been developed in the recent decade for constructing FBG sensors leading to a considerable number of commercial products. To understand the concept of an FBG accelerometer, this work considers a simple linear spring-mass model, in which the FBG accelerometer is the spring system (Figure 2). If the external force causes a displacement Δ*L* of mass *M*, then the strain experienced by the FBG accelerometer can be expressed as:

(4) $$\varepsilon =\frac{\Delta L}{L}=\frac{\Delta \Lambda }{\Lambda }=\frac{1}{P_{e}}\frac{\Delta \lambda_{B}}{\lambda_{B}}$$

where *L* is the effective fiber length. The relationship between the change in fiber length Δ*L* and acceleration *A* is:

(5) $$\Delta L=\frac{M}{k}A$$

where *k* represents the spring constant of the fiber. The external force also induces an internal force in the fiber. Hence, *k*·Δ*L* = *σ·S*, where σ refers to the tensile stress and *S* denotes the cross-sectional area of the fiber. Accordingly, the spring constant can be expressed as:

(6) $$k=\frac{ES}{L}$$

where *E* is the elastic modulus of the fiber. From Equations (4) to (6), the relationship among strain, resonant wavelength shift, and acceleration is:

(7) $$\frac{\Delta \lambda_{B}}{\lambda_{B}}=\frac{P_{e}M}{ES}A$$

This equation reveals that the relative shift of the Bragg wavelength is linearly proportional to the acceleration of the measured system.

**Figure 3.**
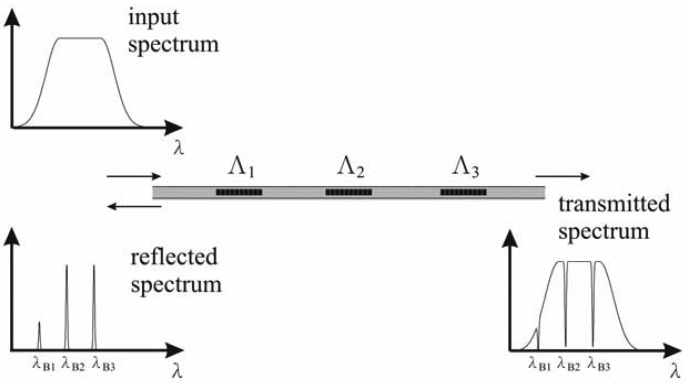
Principle of multiplexing of serial FBG sensors. A sequence of separated FBG sensors with various grating periods, Λ, *i* = 1, 2, and 3, were connected to each other in an optical fiber. The reflected spectrum contains a series of peaks, each associated with a particular wavelength, given by λ*_Bi_* = 2*n*Λ*_i_*.

Multiplexing is an important function of FBG sensors. According to Figure 3, serial multiplexing is performed by connecting a sequence of separated FBG sensors to each other in an optical fiber, each with different grating periods, Λ*_i_*, *i* = 1,2,3,…, *n*. The reflected spectrum contains a series of peaks, each associated with a particular wavelength, given by λ*_Bi_* = 2*n*Λ*_i_*, where λ*_Bi_* and Λ*_i_* are the Bragg wavelength and the *i-th* grating period, respectively. The limit on the number of gratings addressed in this way depends on the width of the source spectrum and the operational wavelength bandwidth required for each grating element [27].

## 3. Setup of a Fiber-Optic Sensing System

**Figure 4.**
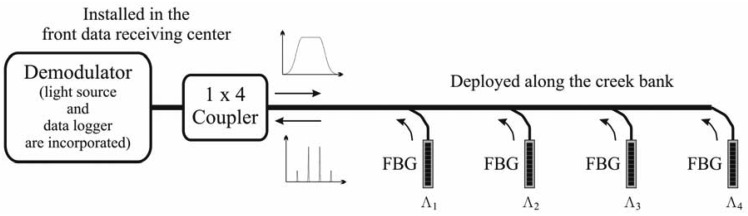
Proposed fiber-optic sensing system for monitoring debris flows. Four FBG accelerometers were installed in a line array along the bank of a creek. Four reflected signals were combined into one using a four-port (1 × 4) fiber coupler and were then sent to the demodulator.

A novel fiber-optical sensing system was established to monitor debris flows in a mountainous region far from available electricity. Figure 4 schematically depicts the proposed sensing system, in which four FBG accelerometers were installed in a line array on a creek bank. Owing to the difference in the frequencies (or wavelengths) of the reflected optical signals from each FBG accelerometer, four reflected signals were combined into one by using the frequency-division multiplexing (FDM) method. The FDM function has been intrinsically incorporated into the demodulator. The associated broadband light source and data logger were also incorporated into a demodulator (BraggSCOPE, FS5500), which was located in the data receiving center (DRC) constructed close to the monitored creeks. Electricity is always available in the DRC. A four-port (1 × 4) fiber coupler was connected to the demodulator in order to either split light from a main fiber into four branch fibers or to combine the four reflected optical signals in the main fiber.

The sensor used here was a commercial single-axis FBG accelerometer (GS6500) with a sensitivity of 10 pm/g and a resonance frequency of 430 Hz. The operating frequency range of this instrument is 0–300 Hz, which range covers the frequencies of ground vibrations generated by debris flows. The measurement range of this instrument was ±40 g, with an accuracy of 20 mg. The operating temperature and humidity were −20 to 80 °C and <90% at 40 °C, respectively. The FS5500 BraggSCOPE, manufactured by Fiber Sensing Company, was located at the data receiving center to provide the broadband light with a wavelength from 1,510 nm to 1,590 nm and store the recorded data at a sampling rate of 500 Hz.

## 4. Calibration Experiments

Performance of the proposed fiber-optic sensing system was compared with those of conventional systems using three-axes geophones (GS-20DX) or microphones (B&K 4190) by conducting field tests to measure the ground vibrations and airborne sounds generated by an artificial debris flow, by using three sensing systems. The operating frequency range of GS-20DX is 8–1,500 Hz with a resonance frequency of 8 Hz and a sensitivity of 27.7 V/(m/s). The B&K 4190 microphone detects sounds with frequencies ranging from 3 Hz to 20 kHz and a sensitivity of 50 mV/Pa. Resonance frequency of the inner diaphragm in B&K 4190 is 8 Hz. The FBG accelerometer and the geophone measure the acceleration and velocity of the ground vibrations, respectively, while the microphone measures the perturbed air pressure.

As mentioned earlier in the Introduction, the characteristics of ground vibrations produced by debris flows are well known. Huang *et al.* [17] compared the ground vibrations with the airborne sounds generated by the impact of rocks against a river bed. Their experimental data revealed that the frequency of both signals ranges from 10–150 Hz and the spatial decay rate of airborne sounds is significantly lower than that of ground vibrations. Their findings suggest that monitoring airborne sounds may be a more efficient means of detecting debris flows. Kogelnig *et al.* [28] reported recently that debris flows generate low-frequency infrasonic signals (4–15 Hz) that can be monitored and correlate with seismic signals.

**Figure 5.**
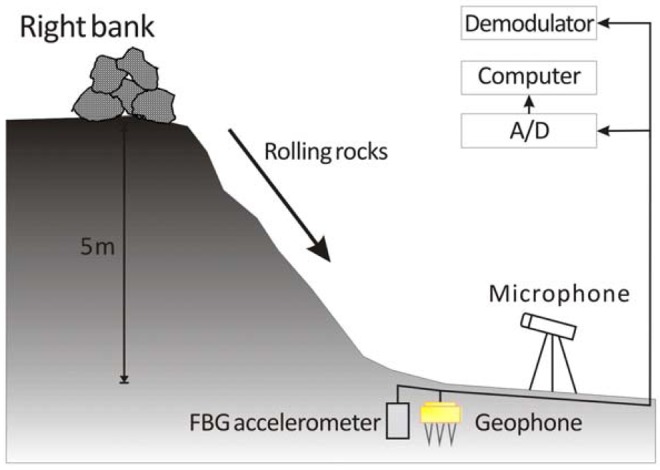
Field test for comparing the performance of the proposed fiber-optic sensing system with those of conventional systems involving a geophone or a microphone.

In this work, the debris flow was simulated by causing rocks and boulders of various sizes to roll down a steep and rugged bank of Chu-Shui Creek in Nantou County, Taiwan. Figure 5 presents the setup of the calibration experiment. A FBG accelerometer and a geophone were buried in the creek bed to detect the ground vibrations caused by this artificial debris flow. The sensors were buried approximately 20 cm below the surface. Both soil and bedrock were filled into the void between sensors and channel bed and, then, pressed to make the test site similar to the surrounding channel bed. A microphone was installed on a large boulder in the neighborhood of the geophone and FBG accelerometer to detect the airborne sounds caused by the artificial debris flow. As is well known, the frequency range of the seismic signals generated by debris flow depends on factors such as rock size, geological conditions, and debris flow type. Based on the work of Huang *et al.* [4] frequencies of ground vibrations caused by motion of rocks are within the frequency range of ground vibrations captured during the debris flow. Therefore, the ground vibrations produced by the artificial debris flow can be assumed to be representative of those associated with a real flow.

**Figure 6.**
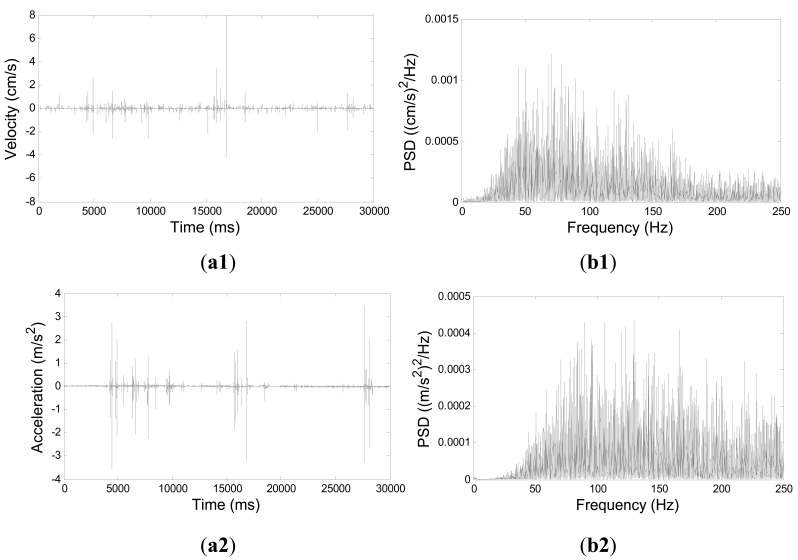
Ground vibrations detected by Z-axis of geophone and FBG sensor; (**a1**) time-series data detected by geophone, (**b1**) geophone signals in frequency domain, (**a2**) time-series data detected by FBG sensor, and (**b2**) FBG sensor signals in frequency domain.

Seismic signals recorded by the geophone in three different axes (X, Y and Z) are approximately the same, where X and Y axes are in the horizontal plane and Z-axis is perpendicular to the ground. For brevity, only the Z-axis signals were analyzed in this work. Figure 6 compares the signals detected by the Z-axis component of geophone and by the FBG accelerometer. This work is concerned with the ground vibration signals with frequencies ranging from 10 to 250 Hz. According to the Nyquist sampling theorem [29], the sampling rate is set to 500 Hz. Because the data loggers of FBG accelerometer and geophone were not the same, the clocks in the two loggers were not synchronized. For synchronizing the signals obtained by these two instruments, cross-correlation was determined to align the two signals by using the first impact pulse. Figure 6(b1) shows the power spectral density (PSD) of the time-series data in the first 30 s of the recorded geophone signals, while Figure 6(b2) shows the PSD of the corresponding FBG sensor signals. Notably, according to Figure 6(b1,b2), the frequency range of the ground vibrations generated by the artificial debris flow was 20–250 Hz based on a sampling rate of 500 Hz. The frequency range of ground vibrations detected by the FBG accelerometer is consistent with that measured by the geophone. However, in Figure 6(b2), the signals at frequencies exceeding 170 Hz have an amplitude comparable with those at lower frequencies. This finding suggests that the FBG accelerometer is more sensitive for sensing tremors of higher frequencies.

The signal-to-noise ratio (SNR, S/N) is usually defined as the power ratio between a signal and the background noise. SNR can be used to evaluate the performance of different sensors [30]. In this work, SNRs for various sensors are computed as follows:

(8) $$\text{SNR}=10\log(\frac{S_{E}}{N_{\text{mean}}})$$

with:

$$S_{E}=\frac{1}{T}\int_{0}^{T}S^{2}(t)dt$$

and:

$$N_{\text{mean}}=\frac{1}{M}\sum_{j=1}^{M}\left[\frac{1}{T}\int_{0}^{T}N_{j}^{2}(t)dt\right]$$

where *S* and *N_j_* denote the recorded time-series signals and background noises, respectively; *S*_E_ represents the mean signal energy in the time interval *T*; and *N_mean_* is the average of *M* samples of noise energy in the same duration *T. T* = 1 s was used and *M* = 20 for determining *N_mean_*. The signals in Figure 6(a1,a2) were used to determine the SNRs of the geophone and the FBG sensor, respectively. Background noises were recorded by the corresponding sensors before the experiments.

**Figure 7.**
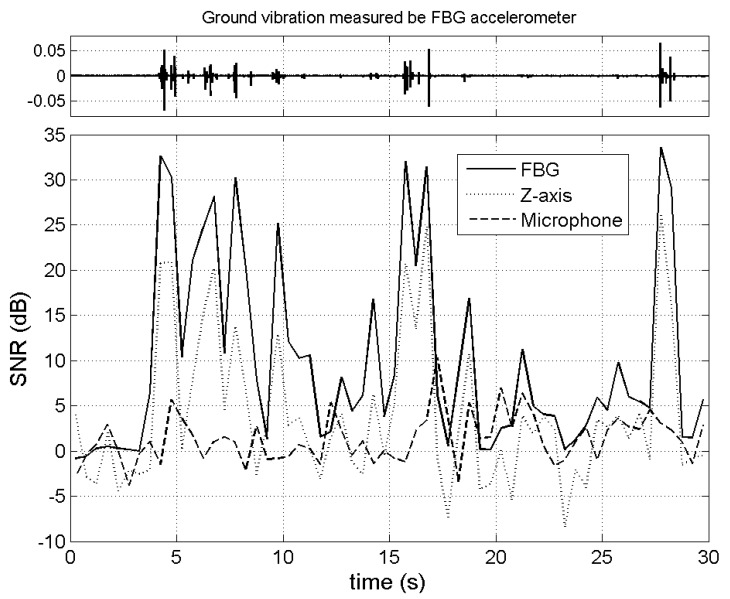
Signal-to-noise ratio (SNR) of signals detected by FBG accelerometer, Z-axis of geophone, and microphone. The upper subplot was the ground vibration signal detected by FBG accelerometer.

Figure 7 presents the SNRs of three sensing systems, indicating that the SNR of the FBG accelerometer exceeds that of the geophone. Above results suggest that the fiber-optic sensing system is more sensitive than the conventional sensing system that uses geophones (*i.e.*, moving-coil sensors). As mentioned earlier, the fiber-optic sensor is also sensitive to variations in the ambient temperature. A future study should clarify the effect of a change in temperature on the recorded signals in order to prevent misinterpretation of the detected signals. Additionally, although the airborne sound measured by the microphone was used as additional information for detecting debris flows, this experiment revealed that the SNR of the microphone was the lowest among the three instruments. This observation may be owing to that the airborne sound generated by the artificial debris flow is smaller than the ambient noise caused by windy weather on the test day.

## 5. Deployment of the Fiber-Optic Sensing System

Having confirmed the capability of the fiber-optic sensing system, this work implemented the proposed system along both Ai-Yu-Zi and Chu-Shui Creeks in Nautou County of Central Taiwan for monitoring debris flows. When the systems were deployed, the fiber length and overall attenuation were estimated using an optical time domain reflectometer (OTDR). OTDR was also used to locate faults, including breaks. For brevity, the tested signals obtained by OTDR are not described here.

**Figure 8.**
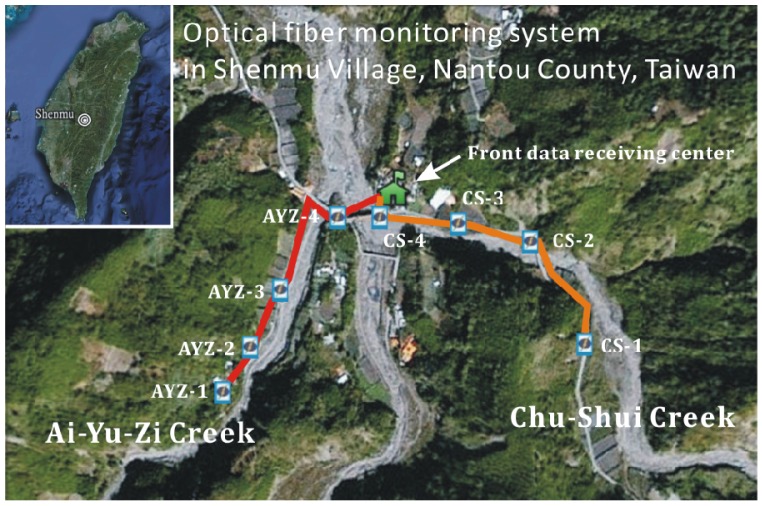
Topographic map and fiber-optic sensing systems deployed along Ai-Yu-Zi and Chu-Shui Creeks of central Taiwan. In each system, four FBG accelerometers were installed along the creek bank. Data were stored in a data logger incorporated into the demodulator, which was located in the front data-receiving center.

Figure 8 displays a topographical map of the study area and the setup of the fiber optical monitoring systems. Table 1 summarizes the distance between the sensors and the distance from Sensor 4 to the data receiving center. Notably, the proposed fiber-optic sensing system is characterized by its low attenuation of the optical signal transmitted in the fiber. In the proposed sensing system, the decay rate was 0.383 dB/km, *i.e.*, markedly smaller than that in metallic conductors (about 5 dB/km) [31,32].

**Figure 9.**
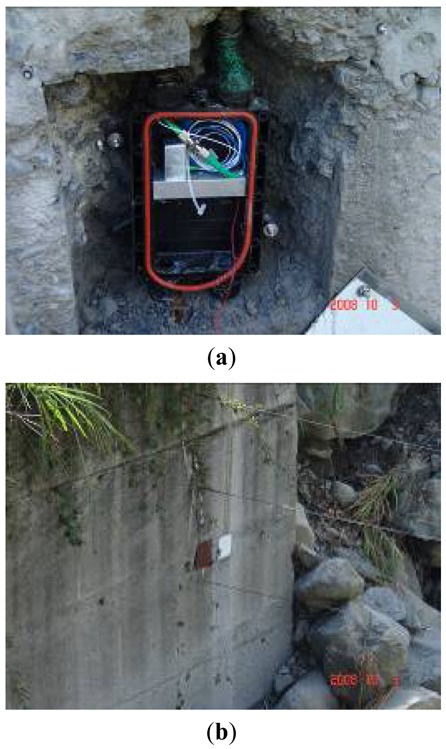
(**a**) Photograph of the FBG accelerometer (the vertical silver one) mounted in a plastic box, which is installed in the bedrock or a check dam to detect the ground vibration caused by debris flows; (**b**) Outlook of the mounted sensors in the bank revetment of Ai-Yu-Zi Creek; left (brown cover): geophone, right (silver cover): FBG accelerometer.

The channel beds of Chu-Shui and Ai-Yu-Zi Creeks are almost always dry, except for during the rainy season or typhoon events. The geological conditions of the two creeks resemble each other. Owing to the debris flow that occurred in August, 2008, rocks, boulders, and soil in the channel beds of Chu-Shui and Ai-Yu-Zi Creeks were loosely packed, explaining why the bed is rugged with randomly dispersed, variously sized rocks. The bedrock of these two creeks consists mainly of sandstone and a shale interlayer. The soil is mainly loam, sandy loam and sand. Figure 9(a) displays a photograph in which the FBG accelerometer is mounted in a plastic box, which is installed in the bedrock or a check dam, to detect ground vibration caused by debris flows. Meanwhile, Figure 9(b) shows the outlook of the mounted sensors (geophone and FBG accelerometer).

**Figure 10.**
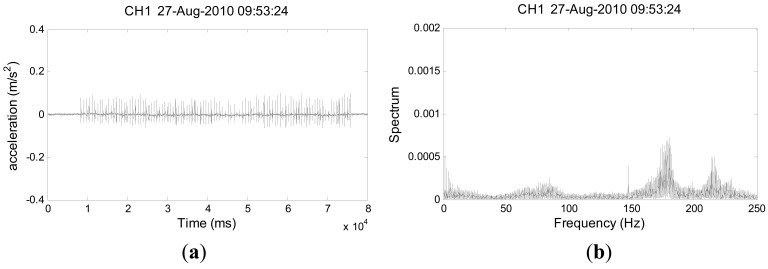

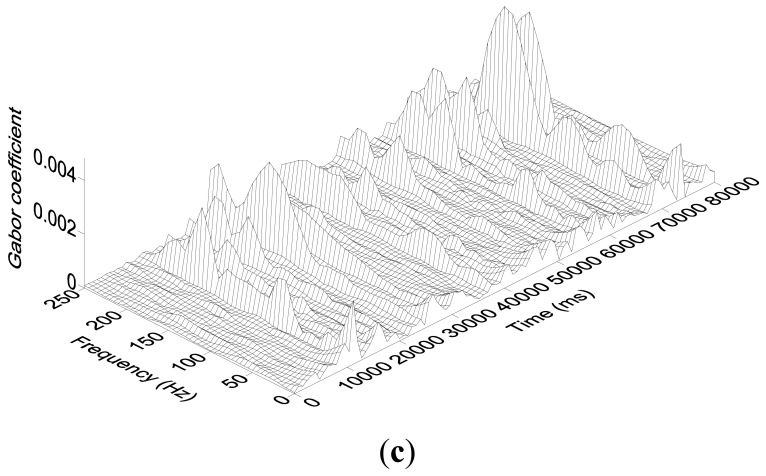
Testing signals measured by Sensor 1 in fiber-optic sensing system deployed along Ai-Yu-Zi Creek (AYZ-1 sensor, Figure 8); (**a**) time domain, (**b**) frequency domain, and (**c**) time-frequency domain obtained by Gabor transform.

This work also tested the sensitivity of the deployed fiber-optic sensing systems along Ai-Yu-Zi and Chu-Shui Creeks through means of generating artificial vibration signals by holding a stone to strike the creek bank continuously where the FBG accelerometer was installed for approximately 1 min. For brevity, Figure 10 presents only one of the typical testing signals. Figure 10(a) plots the time-series data of the vibrations detected by Sensor 1 at Ai-Yu-Zi Creek. The signals produced by each hit of the rock against the bank can be identified. Figure 10(b) shows the signals in the frequency domain, obtained by Fast Fourier Transform (FFT), while Figure 10(c) shows the signals in the time-frequency domain, as obtained by the Gabor transform, which is equivalent to the short-time Fourier transform. The Gabor transform [33–35] was used to transform the seismic signals in the time domain into the time-frequency domain. Notably, the frequency variation of the ground vibration caused by each hit can be identified in detail in Figure 10(c). Furthermore, our observations suggest that the amplitude of accelerations increases with the hit speed and the stone size, and with a decreasing distance between the hit point and the sensor.

This work also demonstrated the multiplexity of each deployed sensing system by generating simultaneously artificial seismic signals near each FBG accelerometer. In the real application of this monitoring system, the original recording data are stored in the data logger of the demodulator; however, the time-averaged acceleration signals were transmitted through the Internet to a remote serving system at a rate of 1 Hz in order to monitor the seismic signals by a PC monitor from a remote site.

## 6. Conclusions

This work describes a novel fiber-optic sensing system for monitoring natural hazards that cause ground vibrations, such as landslides and debris flows. The proposed sensing system consists of a demodulator that incorporates a broadband light source and a data logger, a four-port fiber coupler and four FBG sensors. The ground vibrations generated by debris flows are measured using the FBG accelerometers. Additionally, based on calibration experiments, the performance of the fiber-optic sensing system is compared with those of conventional sensing systems using a geophone or a microphone. Experimental results indicate that the SNR of the FBG accelerometer exceeded the SNRs of the geophone and the microphone.

Following demonstration of the feasibility of the proposed fiber-optic sensing system in detecting ground vibration signals, two sensing systems were deployed along the Ai-Yu-Zi and Chu-Shui Creeks in Nantou County of central Taiwan, to monitor debris flows. In each system, the accelerometers were set up in the creek bank as line arrays. The sensitivity of the established system was also evaluated by hitting the creek bank where the sensors were installed to generate artificial vibration signals. Subsequent data analysis revealed in detail the frequency variation associated with each hit.

Finally, although the sensing capability of an FBG accelerometer appears to be better than that of a geophone, a future study should elucidate how temperature change and other ambient noises affect the identification of seismic signals generated by debris flows.

## Figures and Tables

**Table 1.** Distances between the sensors and the data receiving center (DRC).

**Name of creek**	**Sensor 1-Sensor 2**	**Sensor 2-Sensor 3**	**Sensor 3-Sensor 4**	**Sensor 4 -DRC**
Ai-Yu-Zi	105 m	180 m	196 m	144 m
Chu-Shui	227 m	173 m	145 m	50 m
